# Solar maculopathy secondary to sunlight exposure reflected from the screen of mobile devices: two case reports

**DOI:** 10.1186/s13256-022-03567-5

**Published:** 2022-09-06

**Authors:** Joaquín Marticorena, Ana Honrubia, Javier Ascaso

**Affiliations:** 1 Ophthalmology Department, Hospital Provincial de Conxo, University Hospital of Santiago de Compostela, Rúa Ramón Baltar, s/n., 15706 Santiago de Compostela, Spain; 2 Ophthalmology Department, Hospital Clínico Universitario Lozano Blesa, Zaragoza, Spain; 3 Department of Surgery, School of Medicine, University of Zaragoza, Zaragoza, Spain; 4 Aragon Health Research Institute (IIS Aragon), Zaragoza, Spain

**Keywords:** Albedo, Display, Mobile device, Optical coherence tomography, Solar maculopathy

## Abstract

**Background:**

Solar maculopathy is a well described clinical entity that usually occurs in patients that have gazed directly the sun. In this report we describe the first two cases of solar maculopathy in individuals exposed to sunlight reflected from the screen of mobile devices in the absence of direct sun gaze.

**Cases description:**

*Case 1.* A 30-year-old Caucasic man presented with bilateral metamorphopsia, central scotoma and decreased visual acuity two days after being reading for four hours with his tablet computer in a terrace of a ski center. *Case 2.* A 20-year-old Caucasic woman was examined for bilateral decrease of visual acuity and central scotoma after being at the beach the day before and reading with her mobile phone for 3 hours. Both patients denied gazing directly to sunlight at any moment. In each case, exploration revealed fundus and OCT images compatible with the typical features of solar maculopathy. After 2 years of follow-up, in absence of any specific treatment, *Case 1* had a complete resolution of the fundus alterations, while *Case 2* still presented defects of the outer retinal layers. In both cases, an exposure to sunlight reflected from the screen of their mobile devices was documented in environments where solar radiation is thought to be augmented.

**Conclusion:**

Sunlight reflection from a display screen needs to be considered as a possible risk factor for increased solar radiation and a subsequent risk of solar maculopathy.

## Background

Solar maculopathy is a well described clinical entity that usually occurs in patients that have gazed directly the sun or have viewed an eclipse without the recommended sunlight protection [1]. However, solar maculopathy has also been reported in individuals without a clear history of sun gazing [2-5]. In those patients, particular atmospheric and geographic conditions, such as clear skies or high altitude, are needed to be considered as factors that may increase the amount of solar radiation and subsequent risk of solar maculopathy [6]. In the same way, the presence of snow or sand are known environmental resources of increased light reflectivity. In this report we describe the first two cases of solar maculopathy in individuals exposed to increased sunlight reflected from the screen of mobile devices in the absence of direct sun gaze.

## Cases description

**Fig. 1:**
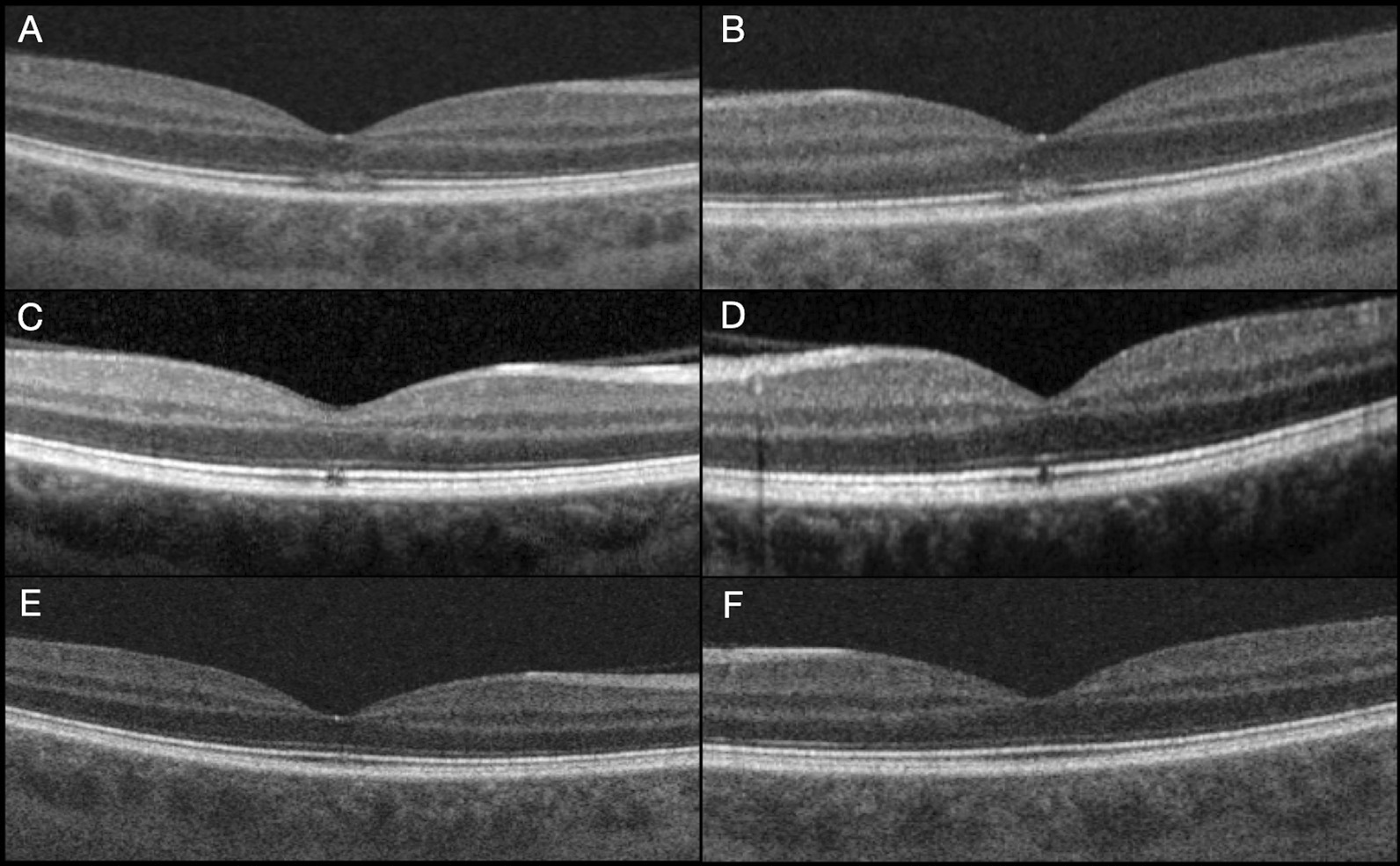
Case 1. **A** (right eye) and **B** (left eye): Initial spectral domain optical coherence tomography image of the right eye with a granular appearance hyperreflective area at the Henle fiber layer (denser at the external retinal layers with disruption of the external limiting membrane, ellipsoidal zone and interdigitation zone at the fovea. **C** (right eye) and **D** (left eye): Month 4 examination with restoration of de external limiting membrane, but persistence of the disruption of ellipsoidal zone and interdigitation zone layers at the fovea. **E** Right eye month 10 examination with a very subtle defect of the ellipsoidal zone, but without a gap. **F** Left eye with complete restoration of all retinal layers after 10 months.

*Case 1.* A 30-year-old Caucasic man presented with bilateral metamorphopsia, central scotoma and decreased visual acuity two days after being reading for four hours with his tablet computer in a terrace of a ski center. At initial examination, best corrected visual acuity (BCVA) was 20/32 in his right eye and 20/25 in his left eye (right eye − 1.00 sph − 0.50 cil 95°; left eye − 1.25 sph). Anterior segment slit-lamp examination was unremarkable. Funduscopy revealed a faint grayish spot at the fovea of both eyes. Red-free photography, blue-light fundus autofluorescence and fluorescein angiography images appeared normal. Optical coherence tomography angiography (OCT-A) (Triton DRI SS-OCTA, Topcon Corporation, Tokyo, Japan) was normal in both eyes. Structural spectral domain optical coherence tomography (SD-OCT) showed a bilateral granular hyperreflective area at the Henle fiber layer (HFL), with disruption of the external limiting membrane (ELM), ellipsoidal zone (EZ) and interdigitation zone (IZ) at the fovea. Considering the clinical, biomicroscopic and tomographic findings, the diagnosis of bilateral solar maculopathy was stablished, despite no direct sun gaze was documented. No specific treatment was given. During the first 4 weeks of follow-up, bilateral central scotoma reduced progressively whereas BCVA improved to 20/25 in his right eye and 20/20 in his left eye. After 2 months BCVA was 20/20 in each eye and remained stable during 2 years of follow-up. Regarding SD-OCT images, a progressive restoration of the outer retinal layers was observed (Fig. 1). No retinal defects were documented on the SD-OCT images after 2 years of follow-up.

**Fig. 2:**
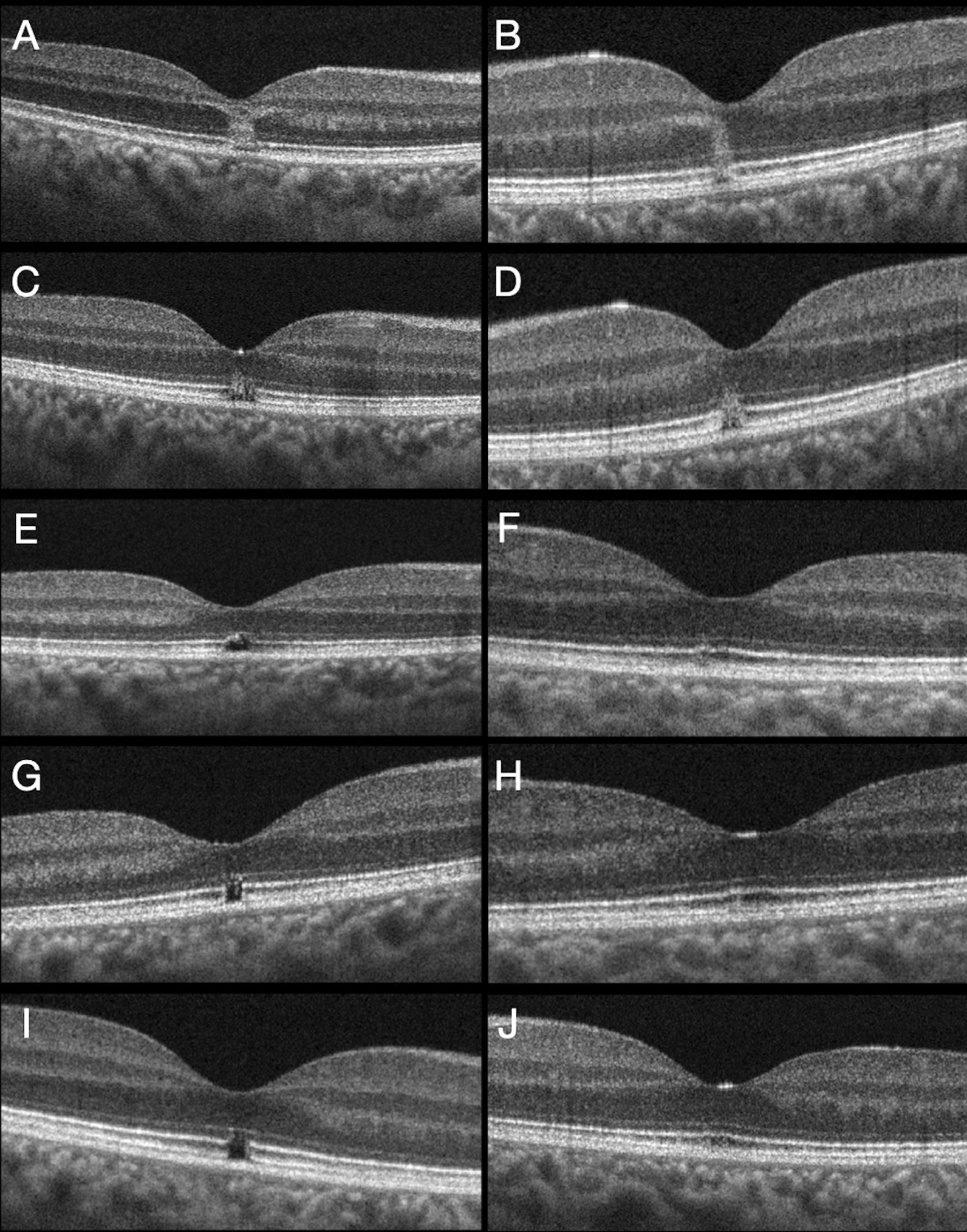
Case 2. **A** Initial right eye structural spectral domain optical coherence tomography scan showing an hyperreflective column extending from the outer plexiform layer to the outer segments of photoreceptors layer with disruption of the external limiting membrane, ellipsoidal zone and interdigitation zone at the fovea. **B** Initial left eye spectral domain optical coherence tomography, with juxtafoveal hyperreflective column extending from the outer plexiform layer to the outer segments of photoreceptors layer with integrity of the external limiting membrane, but with a disruption of the ellipsoidal zone and interdigitation zone. **C** Right eye at 1-week examination showing a reduction of the spectral domain optical coherence tomography hyperreflective column, extending from the outer nuclear layer to the outer segments of photoreceptors layer, with partial restoration of the external limiting membrane, but persistence of the gap at the ellipsoidal zone and interdigitation zone. **D** Left eye spectral domain optical coherence tomography at 1 week with decreased size of the hyperreflective column and disruption of the ellipsoidal zone and interdigitation zone. **E** Right eye 1-month optical coherence tomography with an irregular and partial restoration of the ellipsoidal zone and a interdigitation zone defect at the fovea. **F** Left eye 1 month spectral domain optical coherence tomography with minimal disruption of the juxtafoveal ellipsoidal zone and interdigitation zone layers. **G** Right eye month-5 spectral domain optical coherence tomography with a gap at the foveal ellipsoidal zone and interdigitation zone. **H** Five months left eye examination with restoration of the previous defects at the ellipsoidal zone and interdigitation zone layers. **I** Persistence of the outer retinal defects at the ellipsoidal zone and interdigitation zone of the right eye after 2 years of follow-up. **J** Left eye year-two spectral domain optical coherence tomography with normal appearance.

*Case 2*. A 20-year-old Caucasic woman was examined for bilateral decrease of visual acuity and central scotoma after being at the beach the day before. She denied gazing directly to sunlight at any moment, but referred that she had been reading with her mobile phone for 3 hours without any sunlight protection. At the initial examination, BCVA was 20/63 in her right eye and 20/25 in her left eye (right eye +0.50 sph − 0.50 cil 85°; left eye − 0.50 cil 90°). Slit-lamp anterior exploration was normal. Fundus examination revealed a small yellowish lesion at the macula of each eye, but more prominent in her right eye. Optical coherence tomography angiography (Avanti RTVue XR and Angiovue, Optovue, Fremont, USA) was normal in both eyes. Structural SD-OCT showed a hyperreflective column at the umbo, extending from the outer plexiform layer to the outer segments of photoreceptors layer, with disruption of the ELM, EZ and IZ layers in the right eye. In the left eye a similar, but thinner column was present at the nasal juxtafovea with no defect at the ELM, but with disruption of the EZ and IZ layers. The diagnosis of solar maculopathy was stablished and close following was done in the absence of any treatment. After 1 week, her BCVA remained the same with persistence of bilateral scotoma. A reduction of the OCT hyperreflective column was seen in both eyes, extending only from the outer nuclear layer to the outer segments of photoreceptors layer, with persistence of a clear disruption of the EZ and IZ. At month-1 examination, BCVA was 20/50 in her right eye and 20/25 in her left eye, with recovery in the integrity of the ELM and partial restoration of the disruption of EZ and IZ in the right eye, whereas in the left eye there was a minimal alteration in the integrity of the EZ and IZ layers. After 2 months BCVA was 20/25 in the right eye and 20/20 in the left eye with no significant changes in the OCT images of each eye. At month-5, BCVA was 20/20 in both eyes, but a sensation of small central scotoma persisted in her right eye in correlation with disruption of the EZ and IZ retinal layers, while there were no defects in the retina of the left eye. No functional or anatomical changes were seen after 2 years of follow-up (Fig. 2).

## Discussion

Solar maculopathy is probably an underreported entity since mild cases can produce a temporal decrease in visual acuity with complete functional and anatomical resolution in the absence any specific treatment. Nonetheless, some cases may experience a significant decrease in visual acuity, metamorphopsia and central scotoma. As seen in the OCT images, restoration occurs from the inner to the outer retinal layers. Whereas the hyperreflective area seen in the acute stage of solar maculopathy reduces, the first hyperreflective layer to be restored is the ELM in concomitance with an improvement in visual acuity [1]. The restoration of the outer retinal layers seen in case 1 is compatible with the observation of photoreceptor regeneration if their nuclei has not been affected [7]. However, in some patients the photochemical damage of the outer retinal layers may produce a persistent hyporeflective gap in the EZ and IZ, leading to a cavitation image or outer retina hole, as seen in case 2. This lack of restoration of the outer retinal layers most probably is the result of a permanent loss of photoreceptors [7], which may explain why some patients experience visual deficiencies such as a small persistent central or paracentral scotoma despite having a good visual acuity [8].

In this report, both patients had an asymmetric affection of BCVA, being more severe in their right eye, their dominant eye. In case 2, OCT images clearly revealed a more pronounced initial hyperreflective signal at the fovea of the right eye while it was juxtafoveal and less intense in the left eye. In fact, chronic OCT changes persisted only in the right eye. These differences between the dominant and the non-dominant eye somehow describe that the injure occurred during foveal fixation while they were reading.

Without a clear episode of sun gazing, solar maculopathy needs to be considered as the sum of multiple factors that may favor the exposition of the fovea to an increased amount of short-wave radiation, especially UV-B light. In this report, both subjects were almost emmetropes and with clear crystalline lenses that provided a reduced protection against ultraviolet light [1].

The comprehension of some physical aspects associated with the amount of solar radiation and the behavior of light reflection on the screen of mobile displays is crucial. It is known that solar radiation is increased in higher altitudes and in certain atmospheric conditions such as clear and cloudless sky, warm day and dust and moisture-free sky [6], circumstances that can occur in a ski center or at the beach. It is also important to consider the high albedo (ratio of incident light or radiation that is reflected by a surface) of the surrounding snow and sand surfaces in these two environments [9,10].

In our report, both patients attended to the clinic after having read with their mobile devices for at least 3 hours. It is important to be aware that the tablet computer and mobile phone used were emissive displays. Briefly, the light reflected on the screen of a display is the sum of three components: the mirror-like specular reflection, the diffuse Lambertian reflection and the haze reflection. The mirror-like specular reflection produces a distinct virtual image of the source in the specular direction (i.e. the sun, a light bulb). The luminance of this mirror image is proportional to the luminance of the source. The diffuse Lambertian reflection scatters the incident light uniformly into all directions. The luminance of this scatter is independent of the direction of the illumination and viewing and is proportional to the illuminance received from the light source. The haze appears as a fuzzy ball around the specular reflection. Despite haze reflection has it is own properties, it combines some characteristics of specular reflection and Lambertian reflection. The reflected luminance of the haze is also proportional to the illuminance [11,12]. When the specular reflection difficult the reading on the screen of a mobile device, the user can avoid the reflected light simply tilting the display, but when the source of the disturbing is the diffuse surrounding light, the user tends to increase the brightness of the screen, but still receives the reflected radiation that corresponds to the Lambertian reflection and part of the haze. In outdoor, the different sources of light reflected on the screen were multiple and included direct sunlight, diffuse skylight and light reflected from the surrounding snow or sand. All these sources of light are incoherent and additive in each spectrum of the wavelength [11], including short-wave radiation associated with solar maculopathy.

## Conclusions

As far as the authors are aware, this is the first article describing the presence of solar maculopathy in individuals that were reading on the screen of mobile devices in the absence of direct sun-gazing. Sunlight reflection from a display screen needs to be considered as a possible risk factor for increased solar radiation and the subsequent risk of solar maculopathy. The authors encourage the use of sunglasses with appropriate filter while reading from a display in environments where solar radiation is thought to be augmented.

## Abbreviations

BCVA: Best corrected visual acuity
OCT-A: Optical coherence tomography angiography
SD-OCT: Spectral domain optical coherence tomography
HFL: Henle fiber layer
ELM: External limiting membrane
EZ: Ellipsoidal zone
IZ: Interdigitation zone
OPL: Outer plexiform layer
ONL: Outer nuclear layer

## References

[1] Symons A, Chan H, Mainster MA. Retinal injuries from light: mechanisms, hazards and prevention. In: Schachat AP, editor. Ryan’s retina. 6th ed. China: Elsevier; 2018. p. 1746–56.

[2] Knudtzon K. The prognosis of scotoma helieclipticum. Acta Ophthalmol Copenh. 1948;26:469–94.

[3] Rosen E. Solar retinitis. Br J Ophthalmol. 1948;32:23–35. 10.1136/bjo.32.1.23 PMC510774 18170410

[4] Ridgway AE. Solar retinopathy. Br Med J. 1967;3:212–4. 10.1136/bmj.3.5559.212 PMC1842517 6028468

[5] Shukla D. Optical coherence tomography and autofluorescence findings in chronic phototoxic maculopathy secondary to snow-reflected solar radiation. Indian J Ophthalmol. 2015;63:455–7. 10.4103/0301-4738.159889 PMC4501146 26139811

[6] Yannuzzi LA, Fisher YL, Slakter JS, Krueger A. Solar retinopathy. A photobiologic and geophysical analysis. Retina. 1989;9:28–43. 2717802

[7] Tso MO, Robbins DO, Zimmerman LE. Photic maculopathy. A study of functional and pathologic correlation. Mod Probl Ophthalmol. 1974;12:220–8. 4421002

[8] Garg SJ, Martidis A, Nelson ML, Sivalingam A. Optical coherence tomography of chronic solar retinopathy. Am J Ophthalmol. 2004;137:351–4. 10.1016/S0002-9394(03)00876-6 14962431

[9] Coakley JA. Reflectance and albedo, surface. In: Holton JR, editor. Encyclopedia of atmospheric sciences. UK: Academic Press; 2003. p. 1914–23.

[10] Warren SG. Optical properties of snow. Rev Geophys. 1982;20:67–89.

[11] Kelley E, Jones G, Germer T. Display reflectance model based on the BRDF. Displays. 1998;19:27–34.

[12] Hertel D, Penczek J. Evaluating display reflections in reflective displays and beyond. Inf Disp. 2020;36:14–24.

